# Deep Learning With Data Enhancement for the Differentiation of Solitary and Multiple Cerebral Glioblastoma, Lymphoma, and Tumefactive Demyelinating Lesion

**DOI:** 10.3389/fonc.2021.665891

**Published:** 2021-08-18

**Authors:** Yu Zhang, Kewei Liang, Jiaqi He, He Ma, Hongyan Chen, Fei Zheng, Lingling Zhang, Xinsheng Wang, Xibo Ma, Xuzhu Chen

**Affiliations:** ^1^Department of Radiology, Beijing Tiantan Hospital, Capital Medical University, Beijing, China; ^2^CBSR&NLPR, Institute of Automation, Chinese Academy of Sciences, Beijing, China; ^3^School of Artificial Intelligence, University of Chinese Academy of Sciences, Beijing, China; ^4^College of Medicine and Biological Information Engineering, Northeastern University, Shenyang, China; ^5^Dalian Medical University, School of Stomatology, Dalian, China; ^6^School of Information Science and Engineering, Harbin Institute of Technology at Weihai, Weihai, China

**Keywords:** glioblastoma, lymphoma, tumefactive demyelination, differential diagnosis, deep learning

## Abstract

**Objectives:**

To explore the MRI-based differential diagnosis of deep learning with data enhancement for cerebral glioblastoma (GBM), primary central nervous system lymphoma (PCNSL), and tumefactive demyelinating lesion (TDL).

**Materials and Methods:**

This retrospective study analyzed the MRI data of 261 patients with pathologically diagnosed solitary and multiple cerebral GBM (n = 97), PCNSL (n = 92), and TDL (n = 72). The 3D segmentation model was trained to capture the lesion. Different enhancement data were generated by changing the pixel ratio of the lesion and non-lesion areas. The 3D classification network was trained by using the enhancement data. The accuracy, sensitivity, specificity, and area under the curve (AUC) were used to assess the value of different enhancement data on the discrimination performance. These results were then compared with the neuroradiologists’ diagnoses.

**Results:**

The diagnostic performance fluctuated with the ratio of lesion to non-lesion area changed. The diagnostic performance was best when the ratio was 1.5. The AUCs of GBM, PCNSL, and TDL were 1.00 (95% confidence interval [CI]: 1.000–1.000), 0.96 (95% CI: 0.923–1.000), and 0.954 (95% CI: 0.904–1.000), respectively.

**Conclusions:**

Deep learning with data enhancement is useful for the accurate identification of GBM, PCNSL, and TDL, and its diagnostic performance is better than that of the neuroradiologists.

## Introduction

Cerebral glioblastoma (GBM), primary central nervous system lymphoma (PCNSL), and tumefactive demyelinating lesion (TDL) are distinct neurological lesions with respect to their pathology, treatment, and prognosis. GBM and PCNSL are both malignant primary intracranial tumors in adults (1, 2). The conventional management strategies are surgical resection followed by radiochemotherapy for GBM, and chemotherapy for PCNSL, respectively. The clinical onsets of the two kinds of neoplasms are not specific and closely related to the extent and location of lesions (3). TDL is an inflammatory disease characterized by varied neurodegenerative clinical manifestations, such as movement disorder and vision impairment (4). Hormone therapy is effective for TDL, and the clinical course is more favorable than brain malignancy.

All these three kinds of lesions can be either solitary or multiple (5–7). The conventional MRI findings of these three kinds of lesions are overlapping. As solitary lesions, they usually present as enhanced masses with peripheral edema. As multiple lesions, they present as scattered and enhanced masses in the brain. The open-ring enhancement in TDL may be an important sign that distinguishes it from other tumors (8). This typical radiological finding, however, is not frequent, resulting in a difficult diagnosis.

The similar routine MRI findings represent a challenge for the differential diagnosis. Given the conventional radiology characteristics, some advanced MRI modalities have been used for the differentiation of three lesions. A systematic review showed that the dynamic susceptibility contrast-enhanced image (DSC) and arterial spin labeling (ASL) had the potential to discriminate PCNSL from GBM (9). Another study reported that diffusion-weighted imaging (DWI) could be a useful diagnostic tool to differentiate among PCNSL, GBM, and inflammatory demyelination pseudotumor (10). Moreover, MRS had been a valuable approach to distinguish the mimicked pathologies (11).

However, these advanced MR modalities mainly focused only on the enhanced component of the lesion. Radiomics-based analysis, on the other hand, can explore the whole lesion including the enhanced and non-enhanced components. Recently, different radiomics have been developed to better understand cerebral entities. For example, the deep learning approach has been used for the differential diagnosis or grading in meningioma (12–14).

Thus far, both the advanced MR modalities and radiomics analysis have been used for the differentiation of the three lesions; however, these have only focused on the solitary form of the three lesions. All three lesion types can be multiple (4, 15). Moreover, most radiomics analysis have only considered machine learning algorithms with small datasets (10, 16). Thus, we collected more data and attempted to identify the three types of lesions by using MRI-based deep learning with data enhancement algorithm, with simultaneous focus on single and multiple lesions.

## Materials and Methods

### Ethics Statement

This study is retrospective in nature. It was approved by the ethics committee of Beijing Tiantan Hospital. The need for patient informed consent was waived.

### Subjects

Our study recruited 97, 92, and 72 patients with GBM, PCNSL, and TDL, between January 2005 and December 2019. Of these subjects, 97 patients with GBM, 76 patients with PCNSL, and 52 patients with TDL were from a single medical institute. The remaining 16 patients with PCNSL and 20 patients with TDL were from another medical institute. All patients with GBM and PCNSL were confirmed by pathology. Among 72 patients with TDL, 52 were diagnosed based on the definition of TDL: demyelinating lesions (2 cm or greater) or lesions between 0.5 and 2 cm with possible mass effect that can be mistaken for tumor-like space-occupying lesions and have a characteristic radiographic appearance (17). With analysis of medical records and clinical and MRI characteristics, 20 were pathologically confirmed owing to the diagnostic uncertainty.

The inclusion criteria were as follows: (1) GBM and PCNSL confirmed by pathology, respectively; (2) TDL diagnosed by pathology or the corresponding criteria (17); (3) available cerebral MRI before diagnosis. The exclusion criteria were as follows: (1) patient age <18 years; (2) missing clinical information; (3) receipt of hormone therapy before undergoing MRI; (4) no data on enhanced MRI; (5) lesions not in the cerebral parenchyma; and (6) MR images with obvious artifact. The enrollment process is presented in **Supplementary File 1**. Each type of entity was composed of solitary or multiple lesions.

### MRI Acquisition and Lesions Segmentation

The MRI acquisition protocols were composed of pre- and post-enhanced T1-weighted (CE-T1) images. The contrast media type, venous injection dose, and acquisition parameters for CE-T1 are given in **Supplementary File 2**.

All MR images in the form of digital imaging and communications in medicine (DICOM) were input to the ITK-SNAP (version 3.4.0, www.itk-snap.org). The regions of interest (ROIs) of these three types of lesions were manually delineated on axial CE-T1 by a neuroradiologist using ITK-SNAP.

Before the ROI segmentation, two blinded neuroradiologists with 10 years of experience independently diagnosed these three types of diseases for 130 randomly selected cases out of the 261 cases. Each neuroradiologist had access to the full DICOM images from different MRI scanners. The number of accurately diagnosed cases by the two senior neuroradiologists were divided by that of all diagnosed cases. The result was determined to calculate the diagnostic performance.

### Statistical Analysis

All continuous and categorical variables were expressed as the mean ± standard deviation and the number (percentage), respectively. One-way analysis of variance (ANOVA) and Pearson’s chi-square tests were used to compare the group differences with regard to patient age, sex ratio, and number of multiple lesions by SPSS (version 23.0, IBM Corporation, Armonk, NY, USA). A *p* < 0.05 was considered to indicate statistical significance. The receiver operating characteristic (ROC) curve obtained from the pROC (version 1.16.2) of R (version 4.0.2) was used to show the area under the curve (AUC), accuracy, specificity, and sensitivity under different thresholds to evaluate the performance of the classification model.

### Algorithm Implementation

Most of the algorithms were implemented by Python 3.7.4. The 261 subjects were randomly divided into a training set and a testing set. The training set included 67 cases of GBM, 65 cases of PCNSL, and 50 cases of TDL, and the testing set consisted of 30 cases of GBM, 27 cases of PCNSL, and 22 cases of TDL. All data were converted into a NIFTI format to adapt to a 3D network.

**Figure 1:**
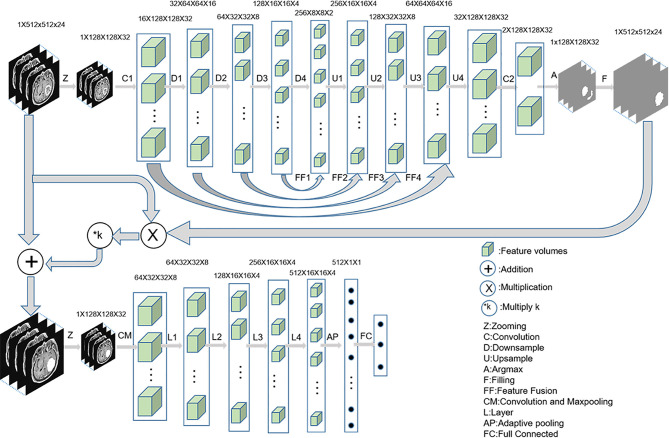
The implementation process of three-stage algorithm based on deep learning with data enhancement. In the first stage, 3D u-net is used to capture the lesion area; in the second stage, enhanced data are generated; and in the third stage, 3D Resnet is used for diagnosis.

The diagnosis process is presented in **Figure 1**. It was divided into three stages and was not an end-to-end solution.

In the first stage, a 3D U-Net (18) was used to automatically predict the lesion area. First, MRI was cropped to reduce the consumption of computing resources, and then the data were normalized to reduce the interference of medical image caused by uneven light. The initial input network image size was reduced to 128 × 128 × 32 without affecting the segmentation performance. Second, the fixed-size image was entered into a convolution layer and four ResBlock downsampling modules to obtain different depth features. Third, the features obtained after the fourth downsampling were fused with the features obtained after the third downsampling module. The fused features were entered into the upsampling ResBlock module to obtain the upsampling features. By analogy, the image size was finally restored to 128 × 128 × 32. Finally, the number of channels were reduced to two after the image entered a convolution layer. The segmentation mask was obtained by argmax function, and the segmentation mask was restored to 512 × 512 × 24 by bilinear interpolation.

In the second stage, the segmented lesion area was combined with the original MR image to change the pixels of the lesion area by a certain multiple, and the pixels of the non-lesion area were unchanged. The following combination equation was used:

Mn=M+M•n•k

**Figure 2:**
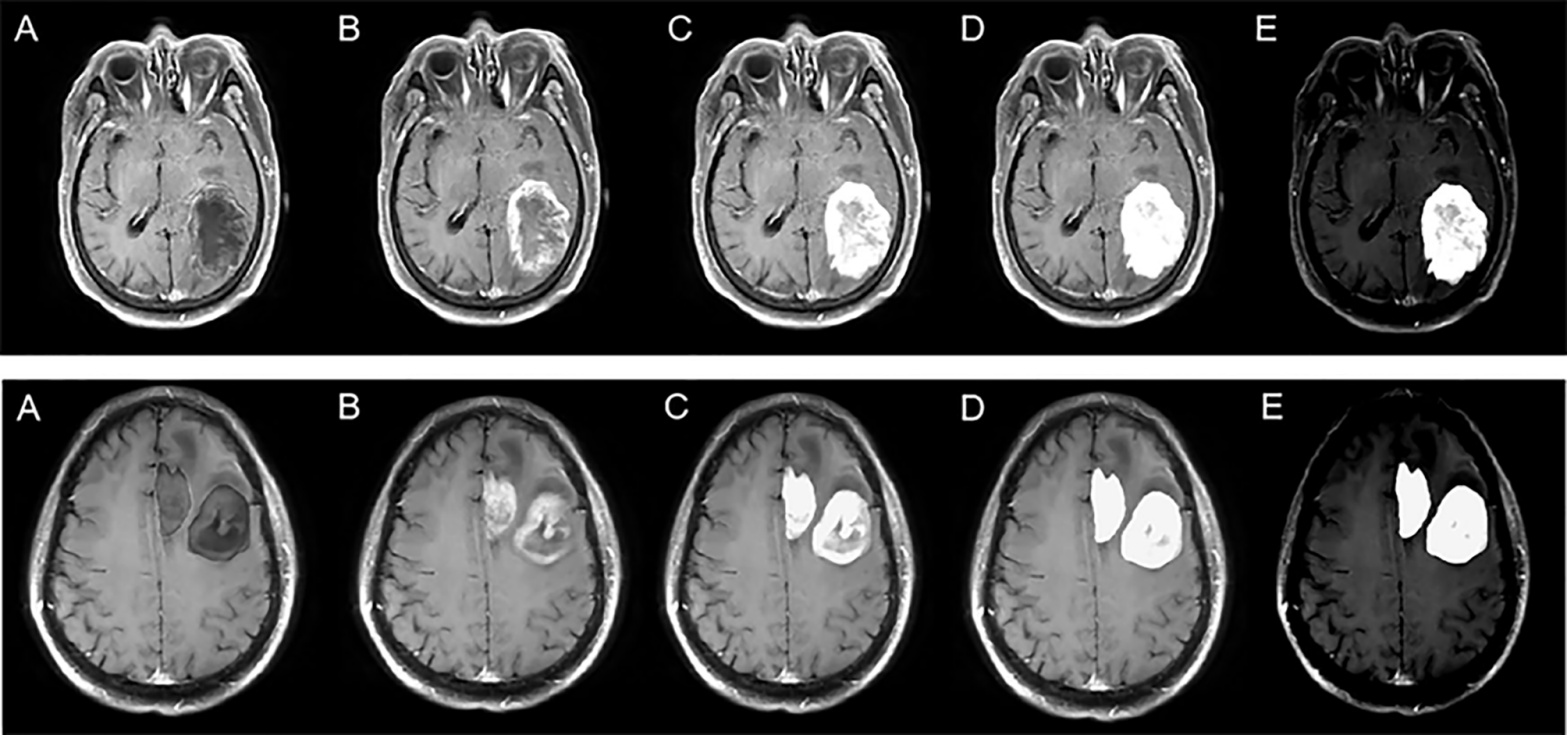
Enhanced data with different k values. The top row represents solitary figures, and the bottom row represents multiple lesions. k is −0.5 **(A)**, 0 **(B)**, 0.5 **(C)**, 1 **(D)**, and 2 **(E)**.

Where *M*n represents the enhanced data, M represents the original MR image, n represents the segmented mask, and k represents the enhancement coefficient. In this experiment, five k values were selected, namely, −0.5, 0, 0.5, 1, and 2, to explore the best model. The *M*n visualization for different k values is shown in **Figure 2**.

In the third stage, the enhanced data were preprocessed similar to the first stage. The Resnet18 (19) was trained and tested by the preprocessed images to identify GBM, PCNSL, and TDL.

**Figure 3:**
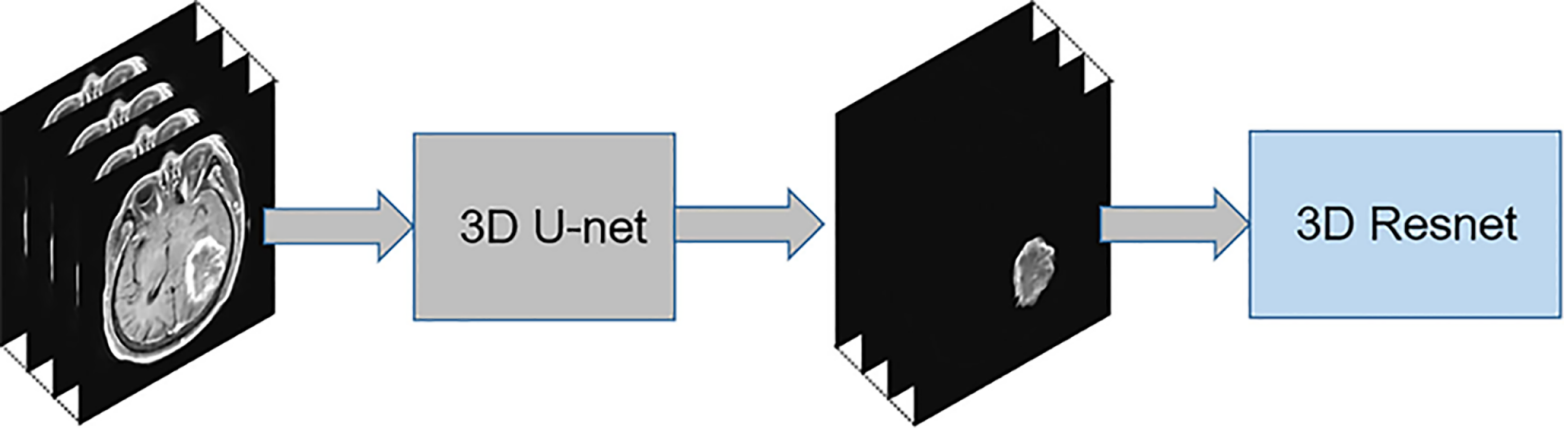
The implementation process of the lesion area diagnosis algorithm.

In addition, identification using the lesion area was considered one of the comparative experiments. The mask segmented by the automatic segmentation network was multiplied with the original MR image so that the surrounding area of the original MR image was removed and only the lesion area was retained. Resnet18 was used for identification of the lesion area. The flow chart of a comparative experiment is shown in **Figure 3**.

The AUC, accuracy, sensitivity, and specificity were calculated according to the output of the classification network. When ROC curves were plotted, one disease was considered positive and the other two were considered negative.

## Results

### Subjects’ Clinical Characteristics

Patients with TDL were the youngest. Those with GBM had the highest ratio of solitary lesions (85.6%, 83/97) (**Table 1**).

**Table 1:** Clinical characteristics of subjects.

	GBM	PCNSL	TDL	*p*
Number of subjects	97	92	72	
Age (years, mean ± SD)	54.61 ± 12.35	53.34 ± 12.57	41.33 ± 12.82	<0.001^*^
**Sex**				0.224
Male	56 (57.7%)	57 (62.0%)	35 (48.6%)	
Female	41 (42.3%)	35 (38.0%)	37 (51.4%)	
Number of lesions				<0.001^*^
Solitary	83 (85.6%)	45 (48.9%)	32 (44.4%)	
Multiple	14 (14.4%)	47 (51.1%)	40 (55.6%)	

*p < 0.05. GBM, cerebral glioblastoma; PCNSL, primary central nervous system lymphoma; TDL, tumefactive demyelinating lesion; SD, standard deviation.

### Diagnostic Performance

**Figure 4:**
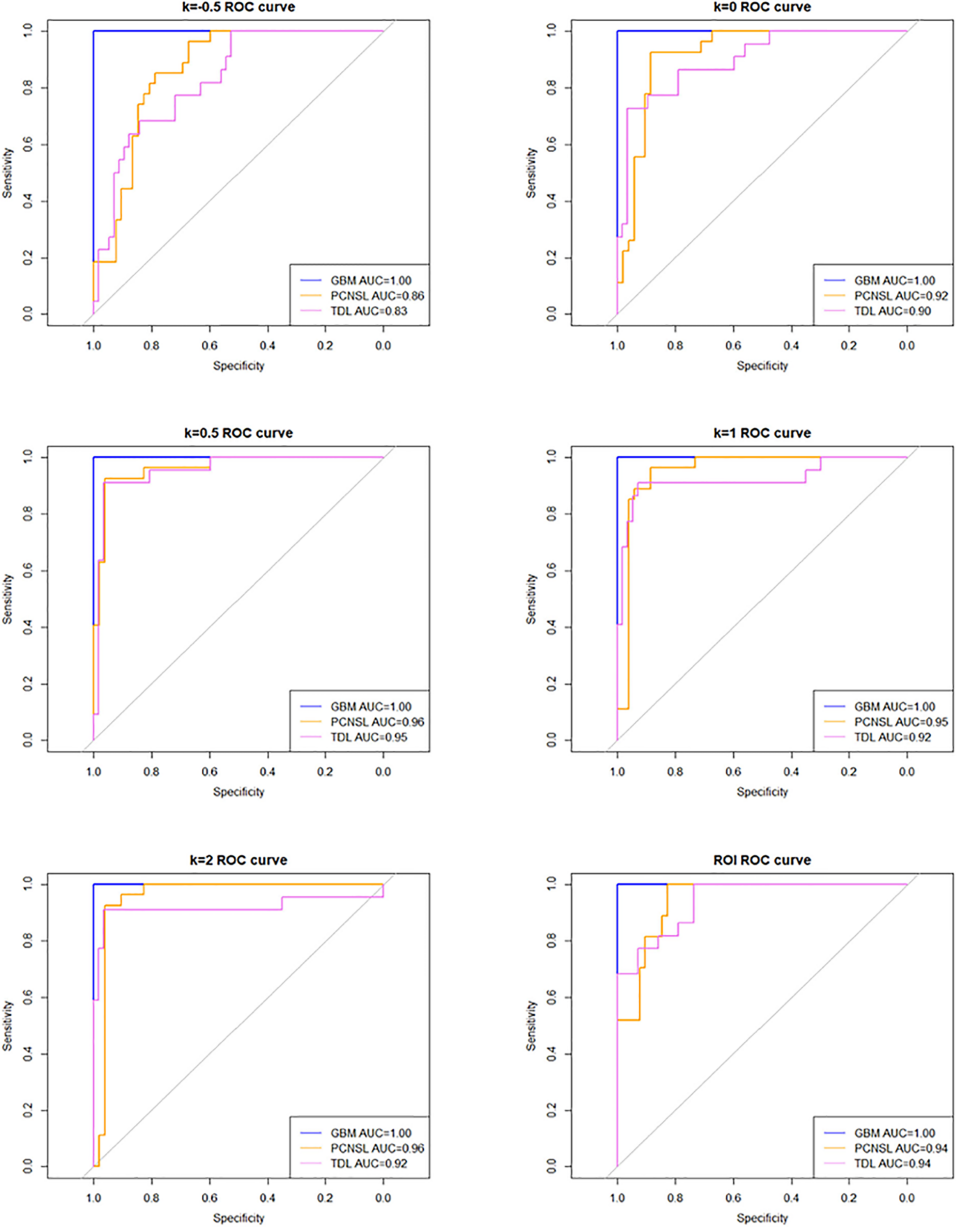
Receiver operating characteristic (ROC) curve at different k values and region of interest (ROI).

**Figure 5:**
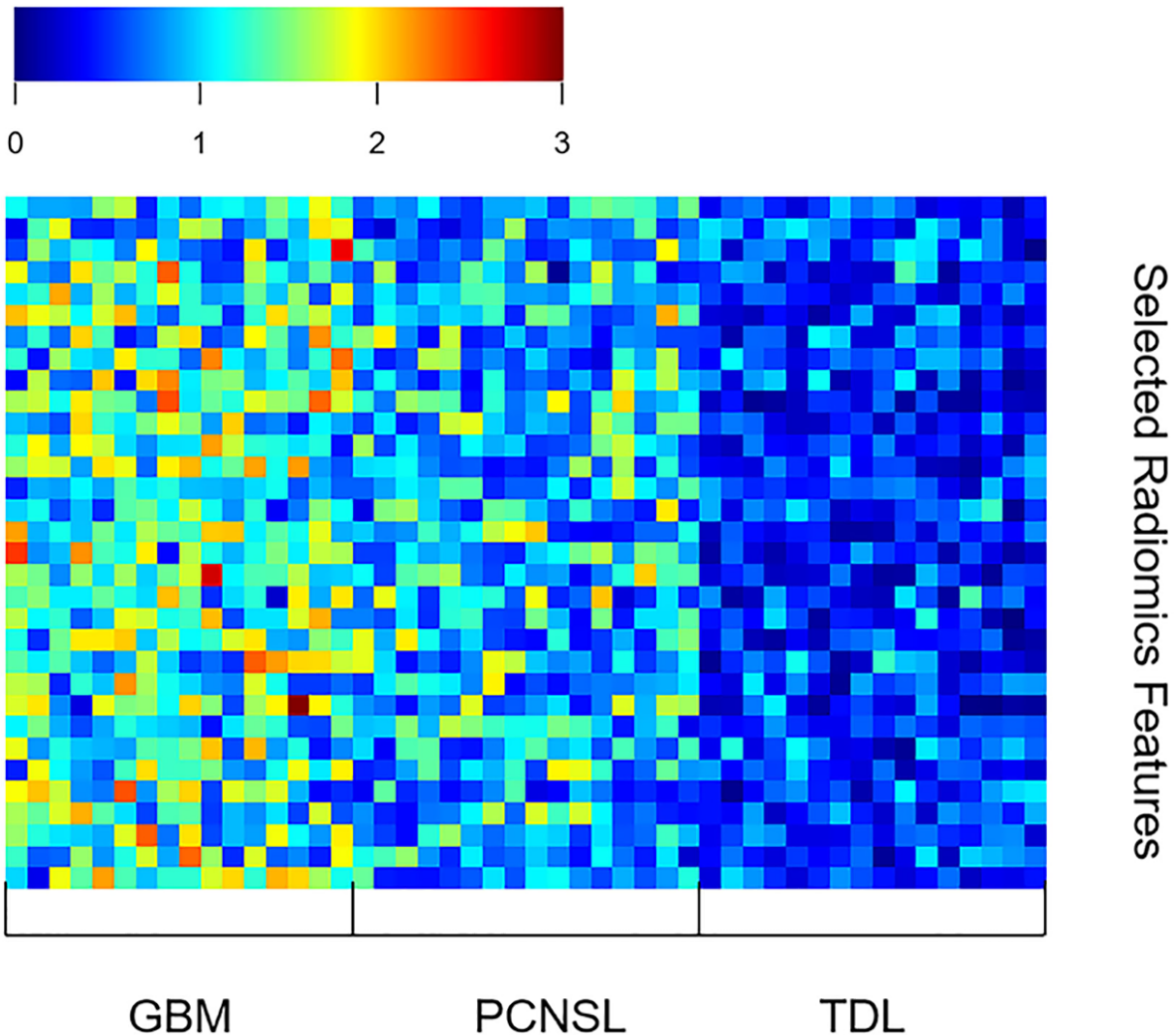
The selected radiomics features of GBM, PCNSL, and TDL at the optimal k value.

The AUC (95% confidence interval [CI]), accuracy, sensitivity, specificity, and overall accuracy are presented in **Tables 2** and **3**. The ROC curves are shown in **Figure 4**. When k was 0.5, the diagnostic performance was the best, and the overall accuracy was 92.4%. The AUC (95% CI) of GBM, PCNSL, and TDL were 1.00 (1.000–1.000), 0.96 (0.923–1.000), and 0.95 (0.904–1.000), respectively. The selected radiomics features of GBM, PCNSL, and TDL at the optimal k value are shown in **Figure 5**. The overall diagnostic performances of the two neuroradiologists were 52.4%.

**Table 2:** Diagnostic performance at different k values.

k	Overall accuracy	AUC (95% CI)			ACC			SEN			SPE		
		GBM	PCNSL	TDL	GBM	PCNSL	TDL	GBM	PCNSL	TDL	GBM	PCNSL	TDL
–0.5	0.81	1.00 (1.000–1.000)	0.86 (0.785–0.943)	0.83 (0.738–0.924)	1.00	0.81	0.81	1.00	0.82	0.55	1.00	0.81	0.91
0	0.86	1.00 (1.000–1.000)	0.92 (0.856–0.980)	0.90 (0.823–0.975)	1.00	0.86	0.86	1.00	0.82	0.73	1.00	0.88	0.91
0.5	0.92	1.00 (1.000–1.000)	0.96 (0.923–1.000)	0.95 (0.904–1.000)	1.00	0.92	0.92	1.00	0.85	0.91	1.00	0.96	0.93
1	0.91	1.00 (1.000–1.000)	0.95 (0.900–1.000)	0.92 (0.838–1.000)	1.00	0.91	0.91	1.00	0.85	0.86	1.00	0.94	0.93
2	0.92	1.00 (1.000–1.000)	0.96 (0.906–1.000)	0.92 (0.813–1.000)	1.00	0.92	0.92	1.00	0.85	0.91	1.00	0.96	0.93

AUC, area under the curve; ACC, accuracy; SEN, sensitivity; SPE, specificity; GBM, cerebral glioblastoma; PCNSL, primary central nervous system lymphoma; TDL, tumefactive demyelinating lesion.

**Table 3:** Diagnostic performance of the model using the lesion area.

	AUC (95% CI)	ACC	SEN	SPE
GBM	1.00 (1.000–1.000)	1.00	1.00	1.00
PCNSL	0.94 (0.900–0.989)	0.84	0.70	0.90
TDL	0.94 (0.892–0.991)	0.84	0.77	0.86
Overall accuracy	0.84			

AUC, area under the curve; ACC, accuracy; SEN, sensitivity; SPE, specificity; GBM, cerebral glioblastoma; PCNSL, primary central nervous system lymphoma; TDL, tumefactive demyelinating lesion.

## Discussion

In our study, GBM and PCNSL were found more in male than female patients, while TDL was found more in female than male patients. This observation was in accordance with previous studies (7, 20). The mean age of patients with these three types of lesions was between 40 and 50 years in our subjects, consistent with some previous reports (7, 20, 21). Regarding the number of lesions, solitary GBM was found in 85.6% (83/97) patients, similar to that reported by Kapoor et al. (6). The ratio of multifocal PCNSL and TDL lesions was slightly higher than that of non-focal lesions, inconsistent with some previous reports (7, 22). This discrepancy may be due to the different case selection criteria among studies.

In this study, we aimed to simultaneously differentiate among three types of lesions. This is different from existing similar studies that only differentiated between two types of lesions. For example, GBM was differentiated from PCNSL by using different MRI modalities (23, 24), machine learning applications (25), and radiomics approach (26); GBM was differentiated from TDL by using methionine positron emission tomography (PET) (27); and PCNSL was differentiated from TDL by using different MRI modalities (28, 29). Moreover, these differential studies between two kinds of entities only focused on solitary lesion. Multiple PCNSL was differentiated from multifocal gliomas by using PET (30). However, the differentiation was also being performed between two types of lesions. To our knowledge, our study is the first to simultaneously differentiate the three entities with solitary and multifocal types by radiomics analysis.

Our study showed that a deep learning algorithm with data enhancement could accurately differentiate among solitary and multifocal GBM, PCNSL, and TDL. This method has two advantages. First, an automatic segmentation network was designed for the lesion region. The neural network can improve focus to the lesion area by enhancing it; this significantly improves the overall diagnostic performance of the neural network for GBM, PCNSL, and TDL. The performance of the model rises first and then falls with the increase in the ratio of lesion area to non-lesion area, and there is an optimal ratio. This means that both the focus area and the non-focus area contain information that can be used for diagnosis. When the ratio is appropriate, the neural network can maximize the information in the two areas for the identification of three lesions. Second, the data were transformed into 3D data, and 3D u-net and 3D Resnet were used for image segmentation and classification, respectively. 3D data have better diagnostic performance than 2D data and 2D networks. The consumption of computing resources is reduced by dividing the training into three stages.

Our study had several limitations. First, although we tried to minimize it, there may be some selection bias owing to the retrospective nature of the study. Second, our training process was not end-to-end, and while this saves computing resources, this makes it more challenging for non-professionals to use this diagnostic method. Although we tried to use the end-to-end network for training, the existing data could not support training several times larger than the existing model to achieve better diagnostic performance. Therefore, more data need to be collected to support end-to-end networks. Third, this experiment only studied the differentiation of three radiologically similar lesions; neuroradiologists may consider additional diseases when making a diagnosis. The existing supervised machine learning and deep learning methods can only diagnose the disease as one of the training set labels, and ignore other possible diseases. If the subject is not one of the training set labels, there is no possibility of being diagnosed correctly. Fourth, no external validation was performed. Last, only single-mode MRI data were used in this study. Inclusion of multimodal data will provide more diagnostic information and is one of the important ways to improve diagnostic performance. However, the single-model data reduce the difficulty of data collection, which makes our method more easily applicable to other diagnosis processes than other methods. Therefore, our method showed good performance in diagnostic accuracy and can provide a feasible reference for the identification of other diseases.

## Conclusion

Deep learning with data enhancement is useful for the identification of GBM, PCNSL, and TDL, and its diagnostic performance is better than that of neuroradiologists.

## Data Availability Statement

The raw data supporting the conclusions of this article will be made available by the authors, without undue reservation.

## Ethics Statement

The studies involving human participants were reviewed and approved by the Department of Radiology, Beijing Tiantan Hospital, Capital Medical University. Written informed consent for participation was not required for this study in accordance with the national legislation and the institutional requirements.

## Author Contributions

XC and XM designed the original research. YZ, KL, JH, HM, HC, FZ, and LZ conducted the research. KL analyzed the data. YZ and KL wrote the manuscript. XC, XM, and XW revised the paper. All authors contributed to the article and approved the submitted version.

## Funding 

This work is supported by the National Key Research and Development Program of China Grant under grant (Nos. 2018YFC0115604, 2016YFA0100900, 2016YFA0100902); the National Natural Science Foundation of China under grant (Nos. 81772005, 82090051, 81871442); Collaborative innovative major special project supported by Beijing Municipal Science & Technology Commission (Z191100006619088); the Youth Innovation Promotion Association CAS (Y201930); and Shandong Province Natural Science Foundation (ZR2020KF016).

## Conflict of Interest

The authors declare that the research was conducted in the absence of any commercial or financial relationships that could be construed as a potential conflict of interest.

## Publisher’s Note

All claims expressed in this article are solely those of the authors and do not necessarily represent those of their affiliated organizations, or those of the publisher, the editors and the reviewers. Any product that may be evaluated in this article, or claim that may be made by its manufacturer, is not guaranteed or endorsed by the publisher.
