# Supplementary material for: Deep Learning With Data Enhancement for the Differentiation of Solitary and Multiple Cerebral Glioblastoma, Lymphoma, and Tumefactive Demyelinating Lesion
Source: Front Oncol. 2021 Aug 18;11:665891. doi: 10.3389/fonc.2021.665891 (PMC8416477; doi:10.3389/fonc.2021.665891)
Supplement: Supplementary file 1 [file DataSheet_1.docx]

**SUPPLEMENTARY MATERIALS**

A

Neuropathological data search in one medical institute between December 20, 2016 and April 1, 2017 using the term “glioblastoma”, n=155

Unavailable preoperative MRI, n=18

MR scanning without contrast enhancement, with obvious artifact, n=13

Patient age<18 years, n=3

Recurrent, with a history of radiotherapy and head operation, n=17

Chemical therapy before MR scanning, n=7

97 consecutive patients

B

Neuropathological data search in two medical institutes between January 1, 2005 and December 31, 2019 using the term “lymphoma”, n=1265

Incomplete clinical information, n=1166

Obvious artifact, n=3

Patient age<18 years, n=4

92 consecutive patients

C

Neurological data search of two medical institutes based on the pathology and corresponding criteria between January 1, 2010 and December 31, 2018 using the term “demyelination”, n=316

Missing clinical information, n=16

Receipt of hormone therapy before undergoing MR, n=123

Patient age<18 years, n=55

No data on enhanced MRI, n=16

Lesions not in cerebral parenchyma, n=34

72 consecutive patients

**SUPPLEMENTARY FILE 1**. The flow chart of the enrollment for patients with GBM **(A)**, PCNSL **(B)** and TDL **(C)**.

After the pre-contrast scanning was finished, the dimeglumine gadopentetate (Beilu Pharmaceutical Co., LTD) was injected into the patient with a dose of 0.2ml/kg via the antecubital venous. Once the injection was finished, the post-contrast scanning started.

| **MRI scanner** | **TR (ms)** | **TE (ms)** | **Slice thickness (mm)** | **FOV** |
| --- | --- | --- | --- | --- |
| GE Discovery MR750 | 2804 | 19 | 5.00 | 512×512 |
| GE Medical System Genesis Signa | 2031 | 19 | 5.00 | 512×512 |
| Siemens MAGNETOM Trio TimSystem | 2000 | 9.8 | 5.00 | 512×432 |
| Siemens MAGNETOM Verio | 1900 | 9.4 | 5.00 | 512×496 |
| Philips Ingenia | 2000 | 20 | 5.00 | 512×512 |

**SUPPLEMENTARY FILE 2**. The scanning parameters for the axial CE-T1 of each MRI scanner.
